# Case report: Multiphenotypic cervical cancer resembling human papillomavirus-related multiphenotypic sinonasal carcinoma

**DOI:** 10.3389/fmed.2024.1507736

**Published:** 2024-12-05

**Authors:** Xiaoxiao Bao, Yan Chen, Jing Zhang, Xiaowei Zhang

**Affiliations:** ^1^Department of Pathology, Affiliated Dongyang Hospital of Wenzhou Medical University, Dongyang, Zhejiang, China; ^2^Department of Gynecology, Dongyang Maternal and Child Health Hospital, Dongyang, Zhejiang, China

**Keywords:** HMSC, HPV, cervical tumor, multiphenotype, case report

## Abstract

Human papillomavirus (HPV)-related multiphenotypic sinonasal carcinoma (HMSC) is a biphasic epithelial tumor associated with HPV infection. This rare tumor primarily affects the nasal cavity and paranasal sinuses, with only two cases reported outside these locations to date—one in the breast and one in the vulva. This report presents a case of a tumor resembling an HMSC arising in the cervix. A 69-year-old female patient presented to our hospital with a history of vaginal bleeding lasting for more than a year. The patient tested positive for high-risk HPV. A cervical biopsy revealed high-grade squamous intraepithelial lesion with invasion. Subsequently, the patient underwent radical hysterectomy for cervical cancer. The postoperative pathological diagnosis, based on histology, immunohistochemistry, and HPV testing, was multiphenotypic cervical carcinoma resembling HMSC. The patient did not receive any adjuvant radiotherapy or chemotherapy postoperatively. Notably, during a year of postoperative follow-up, no recurrence or metastasis was observed. Multiphenotypic cervical carcinoma is a rare tumor that resembles HPV-related multiphenotypic sinonasal carcinoma. These tumors are associated with HPV infection, typically exhibit high-grade histological features, and show a propensity for local indolent progression clinically. Accurate identification of these tumors through their histological characteristics and immunohistochemical staining, followed by confirmation with HPV and molecular genetic testing, is crucial for precise diagnosis, prognostication, and effective management.

## 1 Introduction

Human papillomavirus (HPV)-related multiphenotypic sinonasal carcinoma (HMSC) is a distinct and rare disease entity, newly classified in the 5th edition of the World Health Organization’s classification of head and neck tumors. HMSC is a biphasic epithelial tumor associated with HPV infection, characterized pathologically by surface epithelial and salivary gland-like features. The preferred treatment for HMSC is complete surgical excision; however, adjuvant radiotherapy and chemotherapy may be considered for patients with advanced disease. Most of HMSCs exhibit low-grade malignant biological behavior. HMSC predominantly occurs in the nasal cavity and paranasal sinuses. Notably, to date, only two cases have been reported in other locations—one in the breast and one in the vulva. To the best of our knowledge, we report the first case of a tumor resembling HMSC in the cervix. Additionally, we provide a retrospective analysis of its clinical and pathological features to aid in the diagnosis and treatment of multiphenotypic tumors of the cervix.

## 2 Case description

### 2.1 Case presentation

**FIGURE 1:**
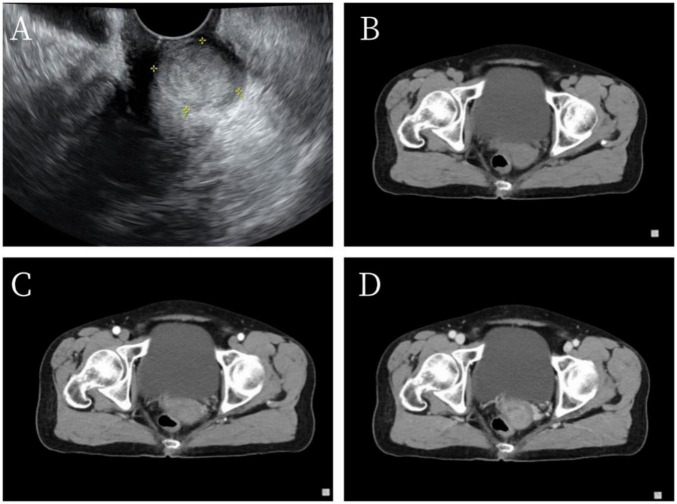
Imaging findings. **(A)** Vaginal ultrasonography indicated a 24 × 19-mm hypoechoic nodule in the posterior lip of the cervix with clear boundaries and heterogeneous internal echoes. **(B–D)** Pelvic computed tomography revealed thickening of the cervical region with persistent enhancement on early- and late-phase scans after contrast administration.

A 69-year-old female patient was admitted to our hospital on June 14, 2023, for surgical treatment of vaginal bleeding that had persisted for more than a year. Initially, the patient experienced minor post-coital vaginal bleeding without associated abdominal pain or hematuria, along with occasional yellowish discharge. Polymerase chain reaction was used to detect 21 HPV genotypes in cervical secretion specimens. High-risk HPV genotyping was positive for HPV types 16, 58, and 42. Transvaginal ultrasonography revealed a 24 × 19-mm hypoechoic nodule in the posterior lip of the cervix, with clear boundaries and uneven internal echoes. This suggested the presence of a cervical leiomyoma (Figure 1A). Contrast-enhanced pelvic computed tomography indicated a slightly enlarged cervix with uniform enhancement and no abnormal density in the adnexal regions. Imaging confirmed slight cervical enlargement with enhancement (Figures 1B–D). The patient had no chronic diseases; no history of surgery, trauma, infectious diseases, or allergies; and did not smoke or drink.

Prior to this hospital admission, a cervical biopsy had been performed on June 7, 2023, in the outpatient department. Pathological examination of the biopsy specimen revealed chronic mucosal inflammation with high-grade squamous intraepithelial lesion and stromal invasion in some areas, consistent with squamous cell carcinoma. A subsequent gynecological examination revealed minor erosive changes on the posterior lip of the cervix. The uterus was atrophic, no significant masses were palpable in the adnexal regions bilaterally, and no tenderness was noted. On June 16, 2023, the patient underwent an open abdominal radical hysterectomy for cervical cancer. During surgery, a 2-cm mass was identified in the posterior lip of the cervix; approximately 3 cm of vaginal tissue and 3 cm of bilateral parametrial tissue were also resected.

### 2.2 Postoperative pathological examination

The uterine specimen measured 10 × 8 × 3 cm, with a thin endometrium and a 1 cm-thick myometrium. The cervical canal was 3 cm in length. A gray-white nodule measuring 2.6 × 2.2 cm was identified within the cervical stroma. The nodule had a moderate texture with areas of friability, and its margins were generally well-defined.

### 2.3 Microscopic findings

**FIGURE 2:**
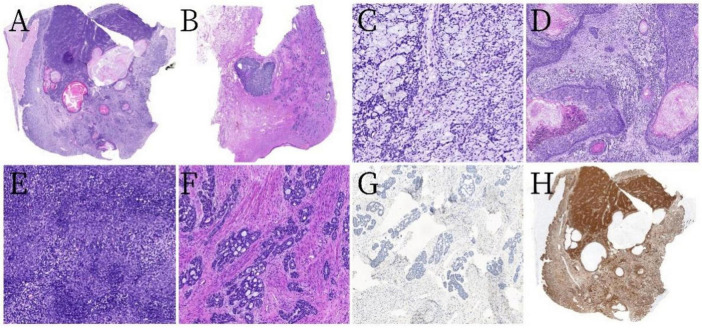
Pathological and histological findings. **(A)** Tumor component one was predominantly diffuse with ill-defined margins, featuring solid sheets and nest-like arrangements in a mosaic pattern, with some areas having relatively clear boundaries (hematoxylin and eosin [H&E] staining at low magnification). **(B)** Tumor component two consisted of solid nodules surrounded by scattered tumor cells distributed in cribriform, tubular, solid small nest-like patterns (H&E, low magnification). **(C)** Mucinous stroma with a sieve-like structure (H&E, ×200 magnification). **(D)** Squamous epithelial differentiation with keratinization and keratin cyst formation with intraepithelial neoplasia (H&E, ×50 magnification). **(E)** Tumor cells were small and arranged uniformly with a high nuclear-to-cytoplasm ratio (H&E, ×200 magnification). **(F)** An abundance of cells in tumor component two arranged in cribriform, tubular, and solid nest-like patterns, with abundant mucin within the lumens and minimal or no stromal reaction (H&E, ×100 magnification). **(G)** The adenoid cribriform area was negative for CD117 by immunohistochemistry (×100 magnification). **(H)** All tumor cells exhibited strong diffuse positivity for p16 (low magnification).

The tumor comprised two distinct components. Component One consisted of diffuse, well-demarcated, solid, sheet-like, nodular tumor masses arranged in a mosaic pattern (Figure 2A). Component Two was characterized by peripheral adenoid cribriform tumor areas surrounding the primary tumor mass (Figure 2B). In Component One, pleomorphic changes were observed, with a background of mucinous, sieve-like structures (Figure 2C). Squamous epithelial differentiation with keratinization and the formation of keratin cysts were noted, along with intraepithelial neoplasia within the squamous epithelium (Figure 2D). Additionally, poorly differentiated areas displayed a diffuse pattern of small, uniform tumor cells with a high nuclear-to-cytoplasm ratio and numerous mitotic figures (Figure 2E). Component Two had cribriform, tubular cells with abundant mucin within the lumen and minimal or absent stromal reactions (Figure 2F).

### 2.4 Immunohistochemical findings

The tumor cells partially expressed both epithelial and basal cell markers. The squamous epithelium, basal-like cell areas, and the adenoid cribriform regions were positive for broad-spectrum cytokeratins (CKs), including CK5/6, CK8, CK19, and squamous epithelial markers (P63, P40). The adenoid cribriform region was negative for CD117 (Figure 2G). In contrast, the poorly differentiated and mucin-rich cribriform areas were diffusely positive for the mesenchymal cell marker vimentin. All the tumor cells exhibited strong diffuse positivity for p16 (Figure 2H). The tumor cells were negative for desmin, smooth muscle actin, S-100, SRY-related HMG-box 10, CD10, PAX8, chromogranin A, synaptophysin, CK7, estrogen receptors, and progesterone receptors. The Ki-67 proliferation index was approximately 60%. Periodic acid-Schiff (PAS) staining was negative.

### 2.5 Pathological diagnosis

The final pathological diagnosis was multiphenotypic cervical carcinoma resembling HMSC. The tumor had infiltrated the deep layer of the cervical stroma but had not penetrated its full thickness. No metastatic cancer was noted in any of the 16 lymph nodes examined. The bilateral parametrial margins and vaginal wall margins were negative.

### 2.6 Follow-up and treatment

The postoperative pathological stage was T1b2N0Mx, and the clinical stage was IB2. Based on these findings, no adjuvant radiotherapy or chemotherapy was administered postoperatively. A 1-year follow-up revealed no evidence of recurrence or metastasis.

## 3 Discussion

In the present case, the morphological characteristics of the patient’s tumor closely resembled those of HMSC, a rare and distinct entity first identified by Bishop et al. (1). HMSC is histologically characterized by a biphasic pattern of surface epithelial and salivary gland-like components. The surface epithelial areas can exhibit pleomorphic changes, including but not limited to ductal, myoepithelial, and squamous differentiation, as well as cellular atypia, hemangiopericytoma-like features, sarcomatous changes, and chondro-osseous differentiation. In salivary glands, HMSC can present as adenoid cystic carcinoma (ACC), basal cell adenocarcinoma, or epithelial-myoepithelial carcinoma, with ACC being the most common. In the present case, the tumor exhibited pleomorphic changes centrally, whereas the peripheral regions displayed adenoid cribriform histology. Immunohistochemically, the diffuse positivity for vimentin in some areas suggested sarcomatous differentiation. The adenoid cribriform region should be distinguished from ACC, and the absence of CD117 expression supports a diagnosis of basal cell adenocarcinoma (2). Cervical tumors that resemble head and neck salivary gland tumors are thought to originate from cervical reserve cells, which can differentiate squamous and glandular epithelium, nevertheless, the presence of myoepithelial differentiation remains uncertain, consequently, it’s frequently noted that cervical glandular tumors resembling salivary gland tumors lack expression of a myoepithelial immunophenotype (3–5).

Additionally, salivary gland-like tumors of the cervix differ from those originating in the salivary glands tumors tend to exhibit extensive necrosis, mitotic activity, solid undifferentiated components, and more pronounced cellular pleomorphism.

The most diagnostically significant feature of HMSC is the detection of high-risk HPV infection, most commonly type 33, though types 35, 56, 16, and 52 have also been reported (6, 7). Recently, two extranasal cases of HMSC have been reported: one in the breast of a 45-year-old woman with HPV types 16, 18, 31, 33, 35, 45, 52, and 58 (8), and another in the vulva of a 47-year-old woman with HPV type 16 (9). In the present case, HPV testing was positive for types 16, 58, and 42. The most significant immunohistochemical finding was strong diffuse positivity for p16.

### 3.1 Differential diagnosis

We considered the following differential diagnoses:

(1) ACC of the cervix: The solid subtype of ACC consists of morphologically uniform basaloid cells arranged in dense nests or sheets within a myxoid or hyaline background, often exhibiting high mitotic activity and occasional tubular or cribriform growth. Immunohistochemically, p16 exhibits strong diffuse positivity, and CD117 is also positive (4). Although ACC shares some features with HMSC in the nasopharyngeal region, it typically lacks pleomorphic changes, and is more aggressive, exhibiting more malignant biological behavior. In this case, pleomorphism was present, CD117 expression was absent, and the clinical course was indolent, ruling out a diagnosis of cervical ACC.
(2) Basaloid squamous cell carcinoma: This carcinoma displays significant atypia and mitotic activity, with diffuse positivity for p63, p40, and CK5/6 (10). However, these markers were only partially expressed in our case. Additionally, basaloid squamous cell carcinoma is more aggressive, with a higher likelihood of metastasis and mortality, making it distinguishable from this case.
(3) Cervical adenosquamous carcinoma: A rare malignant epithelial neoplasm of the cervix that contains clearly identifiable histological components of both adenocarcinoma and squamous cell carcinoma. The two components are intermingled, coexist in transition, or exist in distinct areas, with each component constituting no less than one-third of the tumor (11).
(4) Cervical mucoepidermoid carcinoma: A rare malignant epithelial tumor composed of epidermoid cells, mucous cells, and intermediate cells, with a low incidence rate and a high degree of malignancy. The tumor shows a clear mixture of squamous and mucous cells, with typical intermediate cells visible between them. These tumors are mucin-producing, and diagnosis can be supported through mucin staining with mucicarmine, as well as with PAS and anti-amylase staining (12).
(5) Large cell neuroendocrine carcinoma (LCNEC): LCNEC exhibit marked pleomorphism, enlarged nuclei, and vacuolated chromatin. Immunohistochemistry is crucial for differentiation, as neuroendocrine carcinomas express neuroendocrine markers (13).
(6) Invasive stratified mucin-producing carcinoma of the cervix (ISMC): ISMC exhibits a broad morphological spectrum and cellular diversity, with some cases being mixed-type ISMC (14–16). Most ISMC components, along with stratified mucin-producing intraepithelial lesions, show positive PAS and Alcian Blue staining. In the present case, PAS staining was negative, and the tumor exhibited pleomorphic morphology, allowing for differentiation from ISMC.

Despite exhibiting high-grade histological features, frequent mitotic activity, bone invasion, and common tumor necrosis, HMSCs generally exhibit an indolent clinical course and a favorable prognosis. Treatment typically involves surgical resection, with some patients receiving adjuvant radiotherapy or chemotherapy. A previous study of 38 patients reported 14 cases of local recurrence and only two cases of distant metastasis, one to the lung and the other to a finger (6, 17). To date, there have been no reports of regional lymph node metastases or cancer-related deaths (6). In the present case, the patient with multiphenotypic cervical carcinoma resembling HMSC, clinically staged as IB2, displayed no evidence of recurrence or distant metastasis after 1 year of follow-up.

## 4 Conclusion

This report describes a case of multiphenotypic cervical carcinoma resembling HMSC, which may have originated from cervical reserve cells. The tumor was associated with HPV infection and presented with local progression. Accurate diagnosis, prognostication, and management of such tumors requires histological examination, immunohistochemical staining, and confirmation through HPV and molecular genetic testing.

## Data Availability

The original contributions presented in this study are included in this article/supplementary material, further inquiries can be directed to the corresponding author.
